# Was there a disparity in age appropriate infant immunization uptake in the theatre of war in the North of Sri Lanka at the height of the hostilities?: a cross-sectional study in resettled areas in the Kilinochchi district

**DOI:** 10.1186/1472-698X-12-26

**Published:** 2012-10-31

**Authors:** Ananthan Parameswaran, Pushpa Ranjan Wijesinghe

**Affiliations:** 1 Medical Officer of Health, MOH Office, Moratuwa, Sri Lanka; 2 Consultant Medical Epidemiologist, Epidemiology Unit, 231, De Saram Place, Colombo 10, Sri Lanka

**Keywords:** Conflict, Vaccination, Coverage, Age-appropriate, Sri- Lanka

## Abstract

**Background:**

It was long speculated that there could be under-immunized pockets in the war affected Northern part of Sri Lanka relative to other areas. With the cessation of hostilities following the military suppression of the rebellion, opportunities have arisen to appraise the immunization status of children in areas of re-settlement in former war ravaged districts.

**Methods:**

We conducted a cross-sectional study to describe the coverage and age appropriateness of infant vaccinations in a former conflict district during the phase of re-settlement. The target population comprised all children of re-settled families in the age group of 12 – 23 months in the district. We selected a study sample of 300 children from among the target population using the WHO’s 30 cluster EPI survey method. Trained surveyors collected data using a structured checklist. The infant vaccination status was ascertained by reviewing vaccination records in the Child Health Development Record or any other alternative documentary evidence.

**Results:**

The survey revealed that the proportion of fully vaccinated children in the district was 91%. For individual vaccines, it ranged from 92% (measles) to 100% (BCG, DPT/OPV1). However, the age appropriateness of vaccination was less than 50% for all antigens except for BCG (94%). The maximum number of days of delay of vaccinations ranged from 21 days for BCG to 253 days for measles. Age appropriate vaccination rates significantly differed for DPT/OPV1-3 and measles during the conflict and post-conflict stages while it did not for the BCG. Age appropriate vaccination rates were significantly higher for DPT/OPV1-3 during the conflict while for the measles it was higher in the post conflict stage.

**Conclusions:**

Though the vaccination coverage for infant vaccines in the war affected Kilinochchi district was similar to other districts in the country, it masked a disparity in terms of low age-appropriateness of infant immunizations given in field settings. This finding underscores the need for investigation of underlying reasons and introduction of remedial measures in the stage of restoring Primary Health Care services in the ex-conflict zone.

## Background

Northern and Eastern parts of Sri Lanka, until recently, experienced one of the brutal, long standing civil conflicts in the South-East Asia with severe human and property losses. A significant proportion of the population in the North of the country was internally displaced. Similar to countries where similar, long standing conflict situations prevailed, it is reasonable to believe that the Internally Displaced Population (IDP) did not have adequate access to Primary Health Care (PHC) services due to hostilities between the government forces and rebels [1].

This situation may have affected the vaccination coverage for basic childhood vaccines (National Immunization Schedule at the time of the study is given in Table 1), placing IDPs at a disadvantaged position relative to the general Sri Lankan population which have had a very high vaccination coverage for a considerable period of time [2][3]. Hence, when measures have been taken to resettle them and bring their lives back to normalcy, assessing the status of vaccination, one of the most cost-effective preventive health strategies of vaccine preventable communicable diseases [4], in the re settled population appears to be of paramount importance to the managers of the Expanded Program of Immunization (EPI) in the Northern province with a view to organizing catch-up campaigns and restoration of routine immunization services.

**Table 1:** National immunization schedule in Sri Lanka at the time of the study

**Age**	**Vaccine**	**Remarks**
Preferably within 24 hours of birth	BCG	Vaccination may be given before leaving the hospital or during the first contact with the health services
		If the scar is not present, child may be re-vaccinated after 6 months, up to 5 years of age
On completion of two months	Pentavalent vaccine (DTP- Hepatitis B-Hib ) 1^st^ dose and Oral Polio Vaccine 1^st^ dose	
On completion of four months	Pentavalent vaccine ( DTP- Hepatitis B-Hib ) 2^nd^ dose and Oral Polio Vaccine 2^nd^ dose	
On completion of six months	Pentavalent vaccine ( DTP- Hepatitis B-Hib ) 3^rd^ dose and Oral Polio Vaccine 3^rd^ dose	
On completion of 9 months	Measles containing vaccine 1^st^ dose in the form of Measles vaccine ***	
On completion of one year	Live attenuated Japanese Encephalitis vaccine - SA 14-14-2 **+++**	A single dose is administered
On completion of 18 months	DTP 4^th^ dose and Oral Polio vaccine 4^th^ dose	DTP 4^th^ dose is given in the form of the triple vaccine instead of the pentavalent vaccine
On completion of 3 years	Measles containing vaccine 2^nd^ dose in the form of Measles-Rubella (MR)vaccine ***	
On completion of five years	DT and Oral Polio vaccine 5^th^ dose	Given at the school entry
At 12 years	aTd ( adult Tetanus diphtheria)	Given in the school immunization programme
1^st^ pregnancy	Tetanus Toxoid -1^st^ dose	First dose after 12 weeks of pregnancy and the second dose 6–8 weeks after the first dose
	Tetanus Toxoid – 2^nd^ dose	
2^nd^ pregnancy	Tetatnus Toxoid -3^rd^ dose	After 12 weeks of pregnancy
3^rd^ pregnancy	Tetanus Toxoid -4^th^ dose	After 12 weeks of pregnancy
4^th^ pregnancy	Tetanus Toxoid -5^th^ dose	After 12 weeks of pregnancy
1^st^ pregnancy	Teatnus Toxoid – a booster dose	A booster dose is given if a written evidence of immunization with Tetanus Toxoid containing vaccines according to the National Immunization Schedule is available
Other pregnancies	Tetanus Toxoid –booster dose	If there is a gap of 10 years and more since the last administration of Tetanus Toxoid containing vaccines , a booster is given
***	In the current schedule adopted on 01.10.2011, Measles containing vaccine 1^st^ and 2^nd^ doses have been replaced by Measles-Mumps-Rubella (MMR) combined vaccine.	
+++	In the current schedule Japanese Encephalitis vaccine is administered on completion of 9 months of life while MMR -1^st^ dose is given on completion of one year of life.	

Escalation of the conflict during the period of 2008–2009 had the severest toll on the health of the people mainly in two districts namely Kilinochchi and Mullaithivu. The entire population of these districts was displaced as a result of heavy fighting between government forces and the rebels. The health infra-structure was disrupted, PHC services were discontinued with repetitive displacements of the population and heavy fighting deprived the inhabitants of seeking these services outside their own areas [5-8].This was very much evident in the displaced population within the Kilinochchi district where intense fighting took place. Since, vaccination is believed to be a victim of these circumstances, assessment of its status in the Kilinochchi district in the resettlement phase was a necessity with a view to ensuring equality by eliminating the existing disparity with other regions through planning for restoration of vaccination services.

On the other hand, the displaced population in Kilinochchi is poor and children among them could be malnourished and highly susceptible to communicable diseases [9,10]. Thus, the threat of outbreaks of vaccine preventable diseases looms large unless the vaccination coverage is brought to a particular threshold below which the herd immunity tends to be inadequate to prevent outbreaks. Therefore, appraisals of the vaccination coverage in the Kilinochchi district with a view to introducing catch-up campaigns to cover vulnerable groups are of the highest priority in the resettlement phase.

A child is vaccinated at the earliest age at which he/she is at risk of a disease. The subsequent doses are boosters which are given to maintain the immunity at an optimum level. If the vaccination is delayed, it enhances the vulnerability of children to vaccine preventable diseases [11] and the child is at risk of contracting the disease during that period. Therefore, an assessment of age appropriateness of vaccination is essential to identify delays in routine vaccination, determine the susceptibility of the target population to the disease targeted by the vaccine and correcting delays in vaccination among highly susceptible children in the displaced population currently being resettled in the Kilinochchi district. Moreover, the age-appropriateness of immunization is important to indicate the level of service availability or service utilization [12].

Problems related to access to or utilization of immunization services in the district could be explored by using dropout rates for two vaccines in the schedule. In a stable population, the difference in immunization coverage between two vaccines in the schedule (dropout rate), for instance the first and third DPT/Hepatitis B/OPV, indicates poor utilization of services by the recipients for the subsequent dose, as a higher coverage in the first dose indicates the availability and accessibility of immunization services [13]. Nevertheless, in a migrating population such as the one in Kilinochchi district, the dropout rates may indicate an accessibility problem, utilization problem or both.

Given the above background, we conducted a household survey in the recently resettled population in the Kilinochchi district which was very severely affected by heavy fighting in the last phase of the conflict with a view to describing the vaccination status of infants in resettlements. We specifically focused on the vaccination coverage of infant vaccines and their age appropriateness.

## Methods

We conducted a community based cross-sectional survey in the Kilinochchi district from July 15^th^ to November 15^th^ 2010. The target population comprised all children (toddlers) of re-settled families in the Kilinochchi district in the age group of 12 – 23 months. We selected this cohort of toddlers as they were the immediate cohort of children who were eligible for infant vaccinations within the previous 12 months when the most intense battle took place before the government declared victory over rebels in the Kilinochchi district. Since we focused on toddlers whose families were resettled in Kilinochchi district in the post-conflict resettlement phase as the target population, we decided to exclude from the target population any toddler whose family was originally from another district, not officially re-settled and stayed for a short period (less than 6 months) in the Kilinochchi district. Also excluded was any toddler from another district staying with a re-settled family in Kilinochchi district for any reason. We also excluded 346 families (1% of total families) belonging to Kilinochchi district but still resident in the welfare camps in Vavuniya district. Since we studied the knowledge of parents or legal guardians on infant vaccination as a part of a different study using the same sample, toddlers who did not have parents or a proper legal guardian to collect valid information were also excluded. We selected the study sample from among all children aged 12–23 months resettled in Kilinochchi district (target population) using the World Health Organization’s (WHO) EPI 30 cluster methodology for the household survey [14]. The required minimum sample size for the study was determined using the following formula recommended by the WHO for cluster surveys of immunization coverage [15].

$$n=DE\frac{x\;z^{2}\;x\;p\;x\left(1-p\right)}{d^{2}}$$

In the calculation of the sample size, the Design Effect (DE) was taken as 2. The Standard Normal Deviation (Z) corresponding to the 95% confidence interval was 1.96. The selected degree of precision was 8%.Ideally, it is appropriate to use the reported vaccination coverage as the anticipated proportion (p) of vaccinated children in sample size determination. Though the vaccination coverage reported to the WHO was available for infant vaccinations, there were many concerns about the validity of these figures given the possible over-estimation of the denominator (estimated number of infants) used for calculation of coverage in a conflict affected district with a large number of displacements and within country migrations. The reported coverage for the district was <70%. If 70% was used for sample size determination, the minimum required sample size would have been 252. Having considered issues pertaining to the validity of the reported estimate of coverage, we considered using 50% as the anticipated proportion of immunized children (p) with a view to obtaining the highest possible sample size for the study. Thus, for an anticipated proportion of 50%, the required minimum sample size was estimated to be 300. Since we used 30 clusters to select these 300 study participants, the required number of study participants from a cluster was 10. The lowest administrative division in Sri Lanka is a *Grama Niladhari* (GN) division. We defined a GN division as a cluster. There were 88 GN divisions in Kilinochchi district. From these 88 clusters, thirty clusters were selected Probability Proportionate to their Sizes (PPS). Within the selected 30 clusters, the first house was selected randomly. A recently updated map of the GN division in the resettlement phase was obtained and a pin was dropped on it and the position on the map where the pin pointed was selected as the starting point for selecting the first house.

Once the first house was selected, it was decided beforehand that the immediate house facing the front door and to the right of the first house would be visited. The same principle was followed for subsequent houses. Within the house, interviewers arrayed all children eligible for the study according to their age and selected one study participant randomly. In this manner, houses were visited in all 30 clusters until 10 eligible children were selected from a single cluster. Parents or guardians of selected children were explained the purpose of the research and their written consent for participation was obtained before collecting data. Data collection instruments included a structured checklist in the Tamil language. In the first part, it elicited information on identity of the child. In the second part, it collected information on sex, date of birth and the birth weight of the child. The third part focused on infant vaccines as per the national immunization schedule. In the first column all vaccines due in infancy had been printed. Surveyors used the second column for entering due dates of each scheduled vaccine during infancy. Third column was used for entering the date of the upper limit of the window period from the scheduled date of vaccination so as enable to assess the age appropriateness. Fourth column was used to record the date of vaccination of each infant vaccine. The fifth column was used to indicate if the given vaccine was age-appropriate using the dates recorded in the 3^rd^ column. Sixth column was used to enter the source of information on vaccination [Child Health Development Record (CHDR) or the name of the alternative immunization record], sixth column provided space for entering the place of immunization and the last column indicated if the vaccination was performed during the conflict or post-conflict period. Vaccinations performed before the day of official declaration of ending military activities (19^th^ May, 2009) were entered as those administered during the conflict while those after this date were recorded as vaccinations administered in the post-conflict period.

Tamil speaking, volunteer, high school students waiting for the university entrance undertook the interview in the survey. The team comprised of 60 volunteers, two each per a cluster. They familiarized with research objectives, the check list and techniques of data collection in the class room. Following the training workshop, they were involved in a mock data collection in the field in the adjacent Vavuniya district before independently collecting data. The permission to conduct this study was obtained from the Ethical Review Committee of the Faculty of Medicine of the University of Colombo. No ethical approval was obtained from the region in which the study was conducted.

### Analysis

We summarized binary variables using proportions. These specific proportions were vaccination coverage (%), proportion of age appropriate vaccinations (%) and proportion of invalid vaccinations (%). For each estimate, we calculated 95% confidence intervals.

### Vaccination status

The vaccination status of a child was ascertained using immunization records available in the CHDR or any other alternative documentary evidence. This alternative documentary evidence included any informal vaccination card provided by immunization services run by non-governmental organizations, a medical practitioner or even a state run immunization or hospital paediatric clinic. Immunization status of a child who did not possess a CHDR or any other documentary evidence was recorded as “Not immunized” even if the mother or guardian provided an unrecorded, verbal immunization history.

If the child (toddler) had been given all infant vaccines in the national immunization schedule for the infant period, then the child was categorized as “fully vaccinated ”. If the child had been given one or more than one infant vaccine but not all infant vaccines recommended in the national immunization schedule for the infant period, then the child was categorized as “partially vaccinated ”. If none of the infant vaccines listed in the national immunization schedule was given, then it was categorized as “not vaccinated ”. We used these categories to determine the vaccination coverage of each infant antigen in the district.

### Age appropriateness of immunization

According to the national immunization schedule, each vaccine is given at the completion of a certain age. A particular window period is added to this due date and any vaccination performed on the due date or within the window period is considered as an “age appropriate” vaccination. In this study we used the window period defined by the national immunization programme for determining age appropriateness. According to the national immunization schedule, BCG should be given within 24 hours following the birth. However, BCG vaccination given within 7 days following the birth was considered as an “age appropriate” vaccination. DTP+ Hepatitis B+ Hib and Oral Polio vaccines are given at the completion of 2, 4 and 6 months. If the vaccination was done on the scheduled date or within 2 weeks after the scheduled date, it was considered as an “age appropriate” vaccination. Measles vaccine is due at the completion of 9 months. If it was given on the scheduled date or within 4 weeks after the scheduled date, it was considered as an “age appropriate” vaccination.

### Invalid vaccinations

Vaccinations are considered “valid” when those vaccinations are given when the child reaches the appropriate age. If the vaccination is one of a series (example DTP, OPV), in such an event, when they are given after an appropriate minimum interval of time, these vaccinations are also considered “valid’. Accordingly, we considered vaccinations as invalid when infant vaccinations were given before the scheduled age in the national immunization schedule (table 1) or else they were given before an appropriate minimum interval of time in relation to DTP+ Hepatitis B+ Hib/OPV which are given in a series. The appropriate ages for vaccinating infants with DTP1+Hepatitis B1+Hib1/OPV1, DTP2+Hepatitis B2+Hib2/OPV2, DTP3+Hep B3+Hib3/OPV3, were completion of 2, 4 and 6 months of age respectively. The scheduled age for administering measles containing vaccines was completion of 9 months. Thus, vaccinations administered to the study participants before completing these ages were considered “invalid”. Additionally, if any administration of subsequent doses of the series of DTP+ Hepatitis B+ Hib/OPV had occurred less than 28 days after the preceding vaccination in the series, we considered the current administration as “invalid”.

### Categorization of stages of the conflict

We conducted a bi-variate analysis with a view to identifying the status of infant vaccinations carried out during and after the conclusion of the conflict. In this analysis, we compared the age appropriateness of infant vaccinations by the stage of the conflict. The stage of the conflict was determined using the day of official declaration of the end of conflict (19^th^ May 2009). The period before 19th May 2009 was categorized as “conflict” while the period subsequent to this date was categorized as “post-conflict”. Accordingly, in the bi-variate analysis, we compared proportions of age appropriate vaccinations performed during the conflict and post-conflict stages and tested for statistical significance.

## Results

The study sample consisted of 157 (52.3%) male and 143 (47.7%) female children. The mean age (SD) of study participants was 16.9 months (3.5).

### Immunization coverage

Table 2 shows that the coverage is high for all infant vaccinations in the district. However, this was as low as 92% for the measles vaccine given at 9 months of age. Point estimates and interval estimates have exceeded the Global Immunization Vision and Strategy’s (GIVS) target of over 80% district vaccination coverage.

**Table 2:** Coverage of infant vaccinations in Kilinochchi District

**Vaccine**	**Number vaccinated**	**Number eligible for vaccination**	**Coverage (%)**	**95% CI (%)**
BCG	300	300	100	-
DPT 1+Hepatitis B 1	300	300	100	-
OPV 1	300	300	100	-
DPT 2+Hepatitis B 2	300	300	100	-
OPV 2	300	300	100	-
DPT 3+Hepatitis B 3	296	300	98.7	97.4-99.9
OPV 3	296	300	98.7	97.4-99.9
Measles	275	300	91.7	88.6-94.8

According to the Table 3, the majority (91%) had been given all infant vaccines. Not even a single child who had been given none of the infant vaccines listed in the national immunization schedule was detected in the survey. Among partially vaccinated children (n=27), 85% (n=23) had not been exposed to the measles vaccine given at the completion of 9 months. The observed difference in the proportion of fully vaccinated male (89.8%) and female (92.3%) children as well as the proportion of partially vaccinated male(10.2%) and female (7.7%) children was not statistically significant (p=0.447).

**Table 3:** Infant vaccination status in Kilinochchi district

**Vaccination status**	**Frequency (n=300)**	**Percentage**
Partially vaccinated	27	9 (95% CI=5.8-12.2)
Fully vaccinated	273	91 (95% CI=87.7-94.2)

### Age appropriateness of vaccination

The results in the Table 4 show that the most age appropriate vaccination is for BCG (94%). However, the age appropriateness was low for three doses of DPT +Hepatitis B/OPV. It ranged from 28% for DPT3 + Hepatitis B3/OPV3 to 45.6% for DPT2 / Hepatitis B2/ OPV 2. The age appropriateness of measles vaccination was 49%.

**Table 4:** Age appropriateness of infant vaccinations by antigens in the Kilinochchi district

**Type of vaccine**	**Number (%) vaccinated age appropriately (95% CI)**	**Number (%) vaccinated later than the recommended day**	**Number (%) vaccinated earlier than the due date**	**Number (%) vaccinated, yet dates not available**
BCG	282 (94)	14 (4.6)	0	4 (1.3)
	(95% CI= 91.2-96.6)			
DPT 1+Hep B 1	133 (44.3)	136 (45.3)	9 (3)	22 (7.3)
	(95% CI= 38.6-49.9)			
OPV 1	133 (44.3)	136 (45.3)	9 (3)	22 (7.3)
	(95% CI=38.6-49.9)			
DPT 2+Hep B 2	137 (45.6)	158 (52.6)	0	5 (1.7)
	(95% CI=39.9-51.2)			
OPV 2	137 (45.6)	158 (52.6)	0	5 (1.7)
	(95% CI=39.9-51.2)			
DPT 3 +Hep B 3	83 (28)	211 (71.3)	2 (0.7)	0
	(95% CI=22.9-33.1)			
OPV 3	83 (28)	211 (71.3)	2 (0.7)	0
	(95% CI=22.9-33.1)			
Measles	135 (49%)	110 (40)	2 (0.7)	28 (10.2)
	(95% CI=46-52)			

According to the Table 5, the proportion of infants given the vaccine later than the scheduled date was 45.3%, 52.7% and 71.2% for the 1^st^ , 2^nd^ and 3^rd^ doses of DPT/Hepatitis B / OPV respectively. For the measles vaccination, it was 40%.The mean number of days delayed ranged from 15.4 (SD=4.3) for BCG to 38.9 (SD=3.4) for DPT3/Hepatitis B3/ OPV3. The maximum number of days of delay ranged from 21 days for BCG to 253 days for measles.

**Table 5:** Administration of vaccines later than the recommended date for infants in the Kilinochchi district

**Type of Vaccine**	**Vaccination later than the recommended period**				
	**Number (%) vaccinated late**	**Mean No of days delayed (SD)**	**Median no of days delayed**	**Minimum number of days delayed**	**Maximum number of days delayed**
BCG	14 (4.7)	15.4 (4.3)	16	8	21
DPT 1+Hepatitis B 1	136 (45.3)	20.2 (2.0)	10	1	89
OPV 1	136 (45.3)	20.2 (2.0)	10	1	89
DPT 2+Hepatitis B 2	158 (52.7)	41.2 (4.0)	36	1	179
OPV 2	158 (52.7)	41.2 (4.0)	36	1	179
DPT 3+Hepatitis B 3	211(71.2)	38.9 (3.4)	29	1	115
OPV 3	211 (71.2)	38.9 (3.4)	29	1	115
Measles	110 (40.0)	37.9 (3.7)	30.5	1	253

Table 6 shows that there is no significant difference in the proportion of infants vaccinated with BCG age appropriately during the conflict (91.7%) and the post conflict period (96.1%) (p=0.012). The proportion of children immunized with DPT/Hepatitis B/OPV age appropriately for all three doses during the conflict was significantly higher than the same during the post-conflict (p=0.048,0.01 and <0.001). Paradoxically, the proportion of infants vaccinated with measles vaccine age appropriately during the conflict was lower (36.3%) than that during the post conflict (58.4%) (p<0.001).

**Table 6:** Age appropriateness of infant vaccinations by the stage of conflict

**Vaccine**	**Conflict stage**			
	**Post conflict No (%)**	**Conflict stage No (%)**	**Z value**	**P value**
**BCG**				
Age appropriate	148 (96.7)	134 (93.7)		
Age inappropriate	5 (3.3)	9 (6.3)	0.91	0.34
Total	153 (100)	143 (100)		
**DPT /Hepatitis B /OPV 1**^**st**^**dose**				
Age appropriate	59 (39.9)	74 (57)		
Age inappropriate	89 (60.1)	56 (43)	7.4	0.006
Total	148 (100)	130 (100)		
**DPT /Hepatitis B/OPV 2**^**nd**^**dose**				
Age appropriate	59 (39.1)	78 (54.2)		
Age inappropriate	92 (60.9)	66 (45.8)	6.16	0.013
Total	151 (100)	144 (100)		
**DPT /Hepatitis B/OPV 3**^**rd**^**dose**				
Age appropriate	26 (17.1)	57 (39.6)		
Age inappropriate	126 (82.9)	87 (60.4)	17.42	<0.0001
Total	152 (100)	144 (100)		
**Measles**				
Age appropriate	85 (64.9)	50 (43.1)		
Age inappropriate	46 (35.1)	66 (56.9)	10.92	0.001
Total	131 (100)	116 (100)		

## Discussion

Sri Lanka has enjoyed a high national coverage of vaccination over 90% for vaccines of infancy (BCG, OPV, DTP+ Hepatitis B+ Hib and measles) [16]. In spite of that, specific pockets of under-vaccination were speculated to be existent in the country especially in the areas of civil conflict [2,17]. Contrary to these expectations, our point estimates for coverage of each infant vaccine in Kilinochchi district had exceeded 90% similar to their national coverage [18,19]. Only the lower bound of the 95% confidence interval of measles had been 88.6%. This is consistent with the coverage figures of over 90% reported from other trouble free districts [18,19]. Theoretically, the criterion to exclude toddlers without parents or legal guardians from the sample in our study could have introduced a potential selection bias and led to an over-estimate of the coverage. However, as we did not find any child without parents or legal guardians during enrolment of study subjects in the district, the issue of an over-estimate of coverage did not arise.

Quite interestingly, the official figures of district coverage for these infant vaccines reported to the WHO were less than 70% [18,19]. This reported, low, official coverage may be related to using an over-estimate of the estimated number of births as the denominator for coverage calculations. Since the estimated number of births depends on the population size and the crude birth rate, it could be erroneous due to lack of real population data from the conflict district. As opposed to reported vaccine coverage from many nations with long term conflicts affecting health services, the coverage of fully vaccinated infants in re-settlements in Kilinochchi district was high (91%). For an example, during the war in Bosnia, the overall infant vaccine coverage was reported to be 35% [20]. Nevertheless, similar to Sri Lanka, El Salvador has maintained high vaccination coverage even during conflicts with adequate provision of resources for vaccinations [21].

Though our estimates indicate high vaccination coverage, paradoxically, the cessation of conflict can adversely influence vaccination coverage in the study setting. In other words, disparities of service provision and uptake of vaccines could be potential problems in the post-conflict rehabilitation phase. Ugalde et al. highlighted this phenomenon after the gulf war by pointing out that vaccination services became poor in Gulf due to economic sanctions. Thus, even though the conflict is the underlying cause, the immediate causes affecting vaccination coverage namely availability of services, resources, motivation of the people and their competing priority needs etc. tend to operate during the conflict and are severely aggravated in the post conflict stage [21]. Hence, Sri-Lanka also needs to focus on these factors when vaccination services are re-organized in resettled areas.

The very high health seeking behaviour of Sri Lankan mothers [22], could have contributed to the observed high vaccination coverage even in adverse circumstances. However, there may have been other reinforcing factors that could have contributed to this situation. The health workers of both governmental and international non-governmental organizations in Sri Lanka were aware of the high probability and severe implications of communicable diseases in large concentrations of vulnerable children in conflict situations and preventability of target diseases by infant vaccinations. This could have resulted in organizing systematic, vaccination services leading to an exceptionally high coverage of infant vaccines during the conflict. Additionally, the operational feasibility to organize vaccination service delivery among infants of the displaced population clustered en masse within the district or in surrounding districts would have also contributed to the observed high vaccination coverage.

We found that the dropout rate for the DPT1 and DPT3 in our study was as low as 1.7%. However, with advancing age, the vaccination coverage dropped by 8.3% for measles (9 months) as compared to the 100% vaccination rate for BCG (at birth). It is extremely important, in the current service restoration phase, to identify contributory factors such as accessibility, utilization issues or combination of both for the observed disparity in coverage between vaccines with advancing ages.

This disparity in vaccination coverage between BCG and measles vaccinations impacted on the “fully immunization status “of infants in the district. From the herd immunity point of view also, this dropout rate of 8.3% (95% CI=11.5% -5.8%) for measles will have negative implications for Sri Lanka’s attempt to eliminate measles. It has been suggested that coverage of 90%-94% is needed in England to confer herd immunity against measles [23]. Additionally to the vaccination coverage, it is also dependent on the host resistance too [24]. Therefore a coverage of the above mentioned proportion might be inadequate in a post-conflict situation similar to our study setting where the host potentially could be malnourished. Hence, the susceptibility to measles could potentially be very high in our study setting with drastic implications on measles morbidity and mortality. It invites health authorities to immediately increase the measles coverage to a figure closer to 100% thorough Supplementary Immunization Activities (SIA) for effective and rapid development of population immunity to the required level to eliminate measles in the district. Sustenance of high coverage may be planned in the restoration of vaccination services in newly re-settled areas.

A high age-appropriateness for BCG vaccination in this study was possible and realistic given the low rates of home deliveries in Sri-Lanka even in the theatre of war [25] and high rates of BCG vaccinations given to the new born within 24 hours of the birth or just before the discharge [18,19]. However, the noted delay in BCG vaccinations (6%) for a few babies may have been due to a small number of home deliveries. The other possibility is the possible stock-outs of vaccines in a few hospitals during the conflict.

A remarkable finding in our study was that despite the coverage of over 90% for all infant vaccines, age-appropriateness for each individual vaccine in the schedule of the first year of life excluding BCG was less than 50%. When all infant vaccines were considered, only 9.7% of the infants studied had received all these vaccines age appropriately. In contrast to our findings, the age appropriateness of all infant vaccinations in a group of children aged 12–23 months utilizing exclusively public and private sector immunization providers in the socio-economically strong Colombo district was 92.3% and 95% respectively [26]. This is an ample testimony to the gross disparity existent either in service provision or service utilization or both between our study setting and other areas of the country. In Sri-Lanka, even in areas beyond the theatre of conflict, immunization clinics are usually held once in two weeks. This may result in delayed vaccinations of some children in the event of their next scheduled clinic being missed due to some reasons. Moreover, given the excessive hype caused by increased media reporting of adverse events following immunizations in the country, even for morbidity conditions that are not considered as relative contra-indications, parents and service providers tend to defer vaccinations leading to delayed vaccinations. Nevertheless, the observed low age appropriateness (<50%) for infant vaccines other than BCG and the descriptive statistics of delayed vaccinations in terms of days beyond the window period do not seem to be consistent with above reasons for delay and are more consistent with a marked disparity in frequent and accessible immunization services in Kilinochchi district.

Similar to our findings, a study in the war affected Sierra Leone [11] too has demonstrated that despite a high coverage of vaccination, there could be a delay in vaccination in conflict areas. It underscores that vaccine coverage surveys could be misleading and may mask an underlying potential for outbreaks. This potential is created by the increased number of susceptible children for the target disease at a given point in time due to age-inappropriate (delayed) vaccinations. In Sierra Leone, their increased risk status was further potentiated by the problem of malnutrition and infectious diseases which are prevalent in conflict and post conflict situations [11]. Our findings along with other similar findings in conflict zones underline that delayed vaccination is an aspect that needs serious consideration in theatres of civil wars and conflicts [11,27]. Delayed vaccinations tend to occur in conflict free areas too. However, in stark contrast to a conflict free setting, in conflict settings, delayed vaccinations may put children on additional preventable risk of morbidity and mortality due to the relatively poor child nutritional status and sanitation [11].

Though the conflict is the underlying cause, the existing immediate causes are important for the status of immunization in conflict and post-conflict phases. In contrast to the BCG, we found that the age-appropriateness of DTP + hepatitis B + Hib, OPV vaccinations was significantly higher during the conflict. Several factors may explain the observed difference. When people were displaced in groups and concentrated in particular locations, mostly outside the study area, it was practically feasible for health service providers to capture a large number of eligible infants for vaccination among IDPs. However, situation changed in the post-conflict and re-settlement phase. No longer were IDPs concentrated in particular locations. Nor was it easy for the health services to catch eligible infants scattered over a large geographical re-settlement areas given the scarcity of PHC workers. Moreover, expectations of the re-settled population too would have changed with a situation of competing non-health priorities dominating over health priorities. A heightened stress may also have played a part in significantly higher timely uptake of vaccines during the conflict than in the post-conflict stage [23]. Additionally, the findings may also reflect the lack of availability of services for clients in re-settlement areas where primary health care services are also being gradually restored parallel to human re-settling.

### Limitations

Our study had some limitations. Conducting a cross-sectional study instead of the most appropriate comparative vaccination coverage surveys in the conflict and conflict-free areas is one limitation. The sample size of our study would have been higher, had we used population proportion with specified relative precision rather than using the population proportion with specified absolute precision in calculation of the sample size. Because of the cross-sectional nature of the study, possible misclassification of some toddlers who were vaccinated but did not possess documentary evidence as “non-vaccinated” resulting in an under-estimate of coverage is another limitation. Though we compared vaccinations in the conflict and post-conflict stages, many factors could have influenced the difference in vaccine uptake in two periods. Our study was unable to deal with the differential effect of these factors in conflict and post–conflict settings.

## Conclusions

Though there is no disparity in terms of immunization coverage for infant vaccines, the high vaccination coverage markedly masked the significantly low age-appropriateness of infant vaccinations given in field settings in the war ravaged district in Sri-Lanka. Thus, during conflicts, hostilities may disrupt immunization services and children may receive vaccines at ages later than appropriate. The observed disparity indicates issues related to service availability, service utilization or both. Importantly, the age appropriateness was lower in the post-conflict stage than during the conflict. This finding underscores the need for investigation of underlying reasons and introduction of remedial measures in the stage of restoring PHC services in the ex-conflict zone.

## Abbreviations

EPI: Expanded programme of immunization; PHC: Primary health care; WHO: World Health Organization; CHDR: Child Health Development Record.

## Competing interests

Both authors are public servants employed by the Ministry of Health, Sri Lanka. PRW is attached to the Epidemiology Unit, Sri Lanka which is responsible for determining the policy direction of the National Immunization Programme and helping its implementation through provincial/district health authorities. However, PRW was not involved in any activity related to the immunization programme in the study area. We declare that we have no other conflicting financial interests.

## Authors’ information

**Pushpa Ranjan Wijesinghe** is a Consultant Medical Epidemiologist attached to the Epidemiology Unit of the Ministry of Health. His academic qualifications include MD (General Medicine), MD (Community Medicine/Public Health), MSc (Community Medicine/Public Health) and MPH (Bio-security and Epidemiology).

**Ananthan Parameswaran** is a Medical Officer of Health Moratuwa and has been selected to undergo doctoral training in Community Medicine/Public Health at the Postgraduate Institute of Medicine of the University of Colombo. His qualifications include MBBS (Jaffna), MSC (Community Medicine/Public Health).

## Authors’ contributions

AP designed the study, implemented it in the field, supervised and analyzed the data. PRW designed the study with AP, developed the methodology, contributed to data analysis and prepared the manuscript. Study instruments were prepared jointly by both authors. Both authors read and approved the final manuscript.

## Pre-publication history

The pre-publication history for this paper can be accessed here:

http://www.biomedcentral.com/1472-698X/12/26/prepub
